# Reply to Janssen et al. Comment on “Kremer et al. Kidney Function-Dependence of Vitamin K-Status Parameters: Results from the TransplantLines Biobank and Cohort Studies. *Nutrients* 2021, *13*, 3069”

**DOI:** 10.3390/nu14122440

**Published:** 2022-06-13

**Authors:** Daan Kremer, Dion Groothof, Charlotte A. Keyzer, Coby Eelderink, Tim J. Knobbe, Adrian Post, Marco van Londen, Michele F. Eisenga, TransplantLines Investigators, Leon J. Schurgers, Stefan P. Berger, Martin H. de Borst, Stephan J. L. Bakker

**Affiliations:** 1 Department of Internal Medicine, Division of Nephrology, University of Groningen and University Medical Center Groningen, 9700 RB Groningen, The Netherlands; d.groothof@umcg.nl (D.G.); c.a.keyzer@umcg.nl (C.A.K.); c.eelderink@umcg.nl (C.E.); t.j.knobbe@umcg.nl (T.J.K.); a.post01@umcg.nl (A.P.); m.van.londen@umcg.nl (M.v.L.); m.f.eisenga@umcg.nl (M.F.E.); s.p.berger@umcg.nl (S.P.B.); m.h.de.borst@umcg.nl (M.H.d.B.); s.j.l.bakker@umcg.nl (S.J.L.B.); 2 University Medical Center Groningen Transplant Center, University of Groningen and University Medical Center Groningen, 9700 RB Groningen, The Netherlands; datarequest.transplantlines@umcg.nl; 3 Department of Biochemistry, Cardiovascular Research Institute Maastricht (CARIM), University of Maastricht, 6200 MD Maastricht, The Netherlands; l.schurgers@maastrichtuniversity.nl

We read with interest the comment by Janssen et al. [1] regarding our manuscript [2] on the kidney function-dependence of vitamin K-status parameters. We agree that dephosphorylated uncarboxylated matrix Gla protein (dp-ucMGP) concentrations should be adjusted for kidney function only if changes in kidney function cause changes in dp-ucMGP. We disagree, however, with the argumentation provided by Janssen et al. on why this may not be the case, primarily because Janssen et al. misinterpret the conclusion of a previous study by Rennenberg et al.

Rennenberg et al. calculated the average renal fractional extraction of MGP, as a function of total renal arterial MGP delivery and total renal venous efflux in ninety hypertensive patients. They concluded that the average fractional extraction of MGP is independent of kidney function. The data presented in that article do, however, not allow the conclusion to be drawn that circulating dp-ucMGP concentrations are not kidney function-dependent, like Janssen et al. conclude. Rather, the presented data strongly support the notion that circulating dp-ucMGP concentrations are kidney function-dependent.

First, renal venous MGP concentrations were lower than renal arterial MGP concentrations, which cannot be explained in any other way than presence of a net renal extraction of MGP, either by glomerular filtration, tubular secretion, or both. Importantly, with a molecular mass similar to the emerging glomerular filtration marker cystatin C, and the absence of material renal excretion, which is also quite similar to the behavior of cystatin C, MGP is likely subject to glomerular filtration, followed by tubular uptake and further catabolism without tubular secretion, much like cystatin C [3,4]. One reason the behavior of MGP as a glomerular filtration marker may slightly deviate from cystatin C is that there may be some release of MGP into the renal venous circulation by the kidney, either as a consequence of local intra-renal production, which the data by Rennenberg et al. suggest according to their discussion [5], or incomplete tubular processing and subsequent reuptake of MGP after glomerular filtration.

Janssen et al. are incorrect in their argumentation that the lack of an association between kidney function and fractional extraction invalidates the conclusion that circulating MGP concentrations are kidney function dependent. The data presented by Rennenberg et al. suggest that the extracted percentage of arterially supplied MGP is constant at any level of kidney function [5]. Importantly, any loss of functional kidney tissue is accompanied by proportional decreases in glomerular filtration rate and renal arterial blood (and plasma) flow. There is little variation in filtration fraction over a wide range of rates of glomerular filtration, indicating that glomerular filtration rate and renal blood (and plasma) flow are interdependent. This has been well documented (e.g., in age-related decline of renal function and decrease of renal functional mass related to living kidney donation) [6,7]. Any decrease in kidney function will therefore result in a proportional decrease in renal blood (and plasma) flow, and a concomitant decrease in total arterial MGP delivery to the kidney. Because, according to the conclusions drawn by Rennenberg et al., the percentage of MGP that is extracted from the renal artery remains stable across all levels of kidney function, any decrease in total arterial MGP delivery will lead to a proportional decrease in total MGP extraction, and a consequent increase in the MGP concentration in the circulation. The study therefore suggests that the total amount of glomerular filtration of MGP per unit of time is directly dependent on kidney function, which strongly supports the notion that circulating MGP concentrations are kidney function dependent. Unfortunately, the manuscript of Rennenberg et al. is lacking a scatterplot of circulating MGP concentrations against endogenous creatinine clearance estimated by the Cockcroft–Gault formula. Undoubtedly, such a plot would have been supportive of the conclusion regarding the kidney function-dependence of circulating MGP concentrations.

Moreover, we would like to highlight that in their comment, Janssen et al. only refer to the cross-sectional analyses of our study, which show that kidney function parameters are strongly associated with dp-ucMGP. These findings are in line with a previous study, which showed that a prospective association between dp-ucMGP and incident chronic kidney disease was driven by a cross-sectional association of baseline kidney function with dp-ucMGP concentrations in a large population-based cohort [8]. These findings, combined with our study results [2], are reason for caution in interpreting dp-ucMGP concentrations as a marker of vitamin K status alone [8,9], rather than as a marker that is influenced by both vitamin K status and kidney function.

In addition to the cross-sectional analyses to which Janssen et al. refer, in our article we also show prospective data that further extend the evidence that dp-ucMGP concentrations are kidney function-dependent. In these analyses, we show serial measurements of dp-ucMGP in kidney transplant recipients before and shortly after kidney transplantation [2]. Clearly, the intervention of transplantation improved kidney function, while the included patients underwent no specific vitamin K-targeted interventions. Three months after transplantation, the improvement in kidney function resulted in a decrease in dp-ucMGP concentrations by a median value of 50% (interquartile range: 29% to 63%). This drastic intra-individual decrease in dp-ucMGP after a strong improvement in kidney function by an intervention without any vitamin K-targeted intervention is strong evidence that changes in kidney function cause changes in dp-ucMGP, independent of vitamin K status.

We stress that with the findings from our study, we neither deny potential beneficial effects of vitamin K on the cardiovascular system, nor deny any potential protective effects of carboxylated matrix gla protein on the kidneys through mechanisms suggested by Janssen et al. However, our study reaffirms that dp-ucMGP concentrations are not only strongly dependent on vitamin K status, but also strongly dependent on kidney function, as evidenced by the halving of dp-ucMGP concentrations shortly after kidney transplantation. Contrary to the comment by Janssen et al., this notion is not opposed by the findings previously described by Rennenberg et al., but strongly supported by these findings.

In conclusion, we disagree with the statement in the comment by Janssen et al. that the correlation between dp-ucMGP and creatinine is “far more likely the result of vitamin K deficiency than a function of decreased glomerular filtration rate”. Increased dp-ucMGP concentrations can either be the result of vitamin K deficiency or of disturbed kidney function, or a combination of both. We therefore repeat and further emphasize our recommendation to adequately adjust for baseline kidney function when using dp-ucMGP as a marker of vitamin K status, both in cross-sectional and in prospective, longitudinal studies.
